# Can Zeolite-Supporting Acridines Boost Their Anticancer Performance?

**DOI:** 10.3390/jfb14030173

**Published:** 2023-03-22

**Authors:** Maja Ranković, Anka Jevremović, Aleksandra Janošević Ležaić, Aleksandar Arsenijević, Jelena Rupar, Vladimir Dobričić, Bojana Nedić Vasiljević, Nemanja Gavrilov, Danica Bajuk-Bogdanović, Maja Milojević-Rakić

**Affiliations:** 1 University of Belgrade-Faculty of Physical Chemistry, 11000 Belgrade, Serbia; 2 Department of Physical Chemistry and Instrumental Methods, University of Belgrade-Faculty of Pharmacy, 11221 Belgrade, Serbia; 3 Department of Pharmacy and Center for Molecular Medicine and Stem Cells Research, Faculty of Medical Sciences, University of Kragujevac, 34000 Kragujevac, Serbia

**Keywords:** zeolite, acridine derivatives, drug release, anticancer, cytotoxicity

## Abstract

Acridine and its derivatives (9-chloroacridine and 9-aminoacridine) are investigated here, supported on FAU type zeolite Y, as a delivery system of anticancer agents. FTIR/Raman spectroscopy and electron microscopy revealed successful drug loading on the zeolite surface, while spectrofluorimetry was employed for drug quantification. The effects of the tested compounds on cell viability were evaluated using in vitro methylthiazol-tetrazolium (MTT) colorimetric technique against human colorectal carcinoma (cell line HCT-116) and MRC-5 fibroblasts. Zeolite structure remained unchanged during homogeneous drug impregnation with achieved drug loadings in the 18–21 mg/g range. The highest drug release, in the µM concentration range, with favourable kinetics was established for zeolite-supported 9-aminoacridine. The acridine delivery via zeolite carrier is viewed in terms of solvation energy and zeolite adsorption sites. The cytotoxic effect of supported acridines on HCT-116 cells reveals that the zeolite carrier improves toxicity, while the highest efficiency is displayed by zeolite-impregnated 9-aminoacridine. The 9-aminoacridine delivery via zeolite carrier favours healthy tissue preservation while accompanying increased toxicity toward cancer cells. Cytotoxicity results are well correlated with theoretical modelling and release study, providing promising results for applicative purposes.

## 1. Introduction

Along with novel anticancer drug design emerges a question of their stability, targeted action and favourable efficiency in specific physiological conditions [1]. Due to the often low specificity of drug delivery, there are issues regarding required drug dosage, effective concentration level maintenance over prolonged periods, and healthy tissue-damaging effects. A widely accepted route to overcome these issues is a specific drug carrier platform for sustained delivery [2,3,4], which diminishes the harmful effect that the drug exerts on the healthy tissue, provides dosage control, and attains optimal concentration. In addition to liposomes and various polymeric materials, research on delivery platforms not only focuses on versatile structures with modular performance, but also turns towards a quest for carriers that could boost drug performance [5]. Biocompatibility, low toxicity and documented activity against cancer cells make zeolites perfect candidates for carriers [6,7]. The potential of zeolites in biomedicine relies on their high specific surface with substantial functional centres and tunable structures with different pore sizes that could accommodate a variety of drug structures. If the drug is retained by adsorption on the surface or encapsulation within the pores, a targeted release in the body can be accomplished [8,9,10].

A readily available and medicinally noteworthy aluminosilicate, clinoptilolite, is used as a food supplement and widely tested as a drug carrier [9]. The adsorption capacity of clinoptilolite can be enhanced by functionalization with surfactants, to overcome its unfavorable hydrophilicity [11]. A few adlayers of surfactants may enhance drug loading [4] but, in turn, may lead to instability of the carrier system affecting prolonged release. This is why pharmaceutical recognition of neat and well-defined synthetic zeolites expressing a higher purity degree of crystalline products is expected in the near future. Investigation of detailed interactions in drug/support systems is a crucial prerequisite since it enables particular delivery design and zeolite framework selection to shield healthy cells in the tumour proximity. For this purpose, zeolite nanocarriers with increased permeability are tested for drug encapsulation. Results show reduced or eliminated side effects [12], with retained treatment effectiveness despite lower dosage [13].

It is vital to design drug delivery systems to meet specific criteria for anticancer drugs since this field is a fast-growing one where novel drug propositions are constantly emerging.

Acridine derivatives are nitrogen heterocycles with substituted parent ring structures, exhibiting various biological activities as anticancer, anti-inflammatory, and antimicrobial agents. Acridines can bind nucleic acids by intercalation, influencing many cell processes or interacting with proteins [14]. Thus, acridines have been recently explored as potential anticancer compounds [15]. Acridine derivatives induce tumour suppressor protein transcriptional activity, which stabilization is governed by acridine structure [16]. This is why it is of principal importance to study acridine core interaction with drug carriers. Along with the acridine core, several possible, multi-substituted functional group positions are at hand for interaction with inorganic support/drug carriers. The amino-acridines are potentially important substances with anticancer potential [17], as their amino group enables a number of bonding interactions, making them excellent candidates for zeolite loading.

In addition to the aforementioned amino acridines, the acridine derivatives with potential therapeutic applications are chloroacridines. The electrochemical behaviour of 9-chloroacridine showed that a concentration of 10^−7^ M was the threshold for detecting spontaneous intercalation with DNA [18]. Research in this area continues as new 9-aminoacridine derivatives are synthesized and tested against lung cancer and cervical cancer lines, providing resolved potential for application in cancer treatment [19]. Acridine derivatives, 9-anilinoacridines, are used as topoisomerase II, cancer inhibitor. In this case, a targeted delivery is established through polymer conjugates directly in the tumour tissue with reduced side effects and minimal exposure of the healthy tissue [20]. Another benefit of the carrier system may be seen with acridine-loaded dendrimer supporting the blood-brain barrier pass [21]. Computational studies point to a definite guanine-cytosine/guanine-cytosine (GC-GC) site for 9-aminoacridine while further substitution/functionalization shifts intercalation to the guanine-cytosine/adenine-thymine site GC-AT [22]. The retention time of acridine derivatives at a specific site in DNA affects their biological activity, while higher efficiency is expected at longer retention [23]. This is where the importance of the availability of acridine derivative and its active sites for interaction with cells lies. The accessibility may be significantly enhanced through drug dispersion over solid zeolite carriers compared to bulk [12,24].

Thus, this work aims to (i) prepare acridine and its derivative, a loaded on a faujasite-type structure (FAU) zeolite, (ii) employ spectroscopic and microscopic investigation reinforced by theoretical calculations, (iii) conduct a drug release study, and (iv) investigate the cytotoxicity potential of the prepared samples toward cancer cells and fibroblasts. The selection of the zeolite framework was supported by our recent cytotoxicity study [25,26].

## 2. Experimental Section

### 2.1. Materials

Y zeolite with FAU structure (CBV 780, SiO_2_/Al_2_O_3_ = 80, specific surface area of 780 m^2^/g) was purchased from Zeolyst International and used as is. Tested bioactive molecules are acridine (denoted as Acr, 97%, M = 179.22 g/mol, Sigma Aldrich, St. Louis, MI, USA) and derivatives 9-chloroacridine (9ClAcr, M = 213.662 g/mol, Sigma Aldrich) and 9-aminoacridine (9NAcr, M = 194.232 g/mol, Sigma Aldrich). The impregnation solutions were prepared in 96% ethanol (MilliporeSigma, Burlington, MA, USA).

Drug supporting procedure involved suspension preparation comprising 10 mL of target drug (1 mM) and 100 mg of zeolite, thermostatted at a laboratory shaker at 23 °C/110 rpm (OLS Shaking Bath, Grant Instruments, Cambridge, UK) for 24 h. Suspensions were centrifuged at 13,400 rpm (Minispin, Eppendorf, Hamburg, Germany). The prepared drug-loaded zeolites are denoted as Acr/Y, 9ClAcr/Y and 9NAcr/Y, respectively.

### 2.2. Methods

After adsorption and centrifugation, the amount of drug remaining in the solution was measured at the Agilent Cary Eclipse Fluorescence Spectrophotometer. An excitation wavelength of 356 nm was chosen for Acr, 386 nm for 9ClAcr and 402 nm for 9NAcr. The excitation aperture was 2.5 nm for Acr and 9NAcr, and 5 nm for 9ClAcr, while the emission aperture for all measurements was set at 5 nm. Optimization of experimental conditions due to sensitive fluorescent detection was performed with calibration curves setting at 0.999 correlation coefficients.

The Fourier transform Infrared (FTIR) spectra of samples were recorded with an iS20, Nicolet spectrometer (Thermo Scientific, Waltham, MA, USA), in the range from 4000 to 400 cm^−1^ with 2 cm^−1^ resolution employing the KBr pellet technique. Samples were dispersed in KBr in the amount of 0.7% and 2.7 wt.%. The Raman spectra were recorded using a DXR Raman microscope (Thermo Scientific) using the 780 nm laser, with a 24.0 mW power output, 10× objective and 25 μm pinhole aperture. Ten exposures were made for each spectrum with 10 s exposure time. The automatic fluorescence correction was performed using the OMNIC software (Thermo Scientific).

Drug-loaded surface composition and morphology were tested using a Phenom ProX microscope coupled with an energy-dispersive X-ray spectrometer (EDS) (Thermo Scientific). SEM-EDS was performed at 5000, 10,000, and 20,000× magnification by using the highest acceleration voltage of 15 kV. Selected SEM micrographs were the best resolution for 10,000× magnification, while elemental maps were recorded at 20,000× magnification. Surface mapping required 128 passes with 10 ms dwell time per point.

To mimic the physiological environment of the colorectal carcinoma cells, a phosphate buffer pH 7.2 was used for the drug release study. After suspending 5 mg of drug-loaded zeolite sample in 5 mL or 10 mL of buffer solution, a leached drug quantity was recorded within a 2 h release period. The dissolution kinetics were monitored for 5 mg/5 mL and 5 mg/10 mL suspensions within the timeframe of the cytotoxicity study, 48 h.

### 2.3. Theoretical Calculation

MarvinSketch (Chemaxon, Budapest, Hungary) software was used for pK calculations of investigated acridines. MOPAC2016 computer code was used to perform semi-empirical calculations [27]. PM7 method [28] with complete structural relaxation was carried out. The presence of water as a solvent molecule was modelled implicitly, using the conductor-like screening model (COSMO) method [29].

The crystal structure of FAU zeolite was adopted from the International zeolites association [30] for modelling the interactions with acridines. The crystal structure was described using a primitive rhombohedral unit cell to reduce the number of elements in the model. To fit the Si/Al ratio with one used in experiments, one Si atom was replaced by an Al atom in each of the ring openings. The position of acridines within the zeolite network was optimized while keeping the zeolite fixed.

### 2.4. Cytotoxicity Study

In vitro cytotoxicity test employed the methylthiazol tetrazolium (MTT) colorimetric technique [31] against cell line HCT-116 (human colorectal carcinoma) and MRC-5 fibroblasts, and was performed in triplicate. The cells were incubated with the medium alone or with a two-fold serial dilution of drug-loaded samples and pristine Y zeolite suspensions (5 mg/mL). The cells were incubated under a CO_2_ atmosphere (5%) for 48 h at 37 °C. MTT was dissolved (5 mg/mL) in phosphate buffer saline having a pH of 7.2 and filtered through a 0.22 μm Millipore filter before use. The absorbance was measured at 595 nm on Zenyth 3100 Multimode detector.

## 3. Results and Discussion

### 3.1. Morphology and Surface Elemental Maps

**Figure 1:**
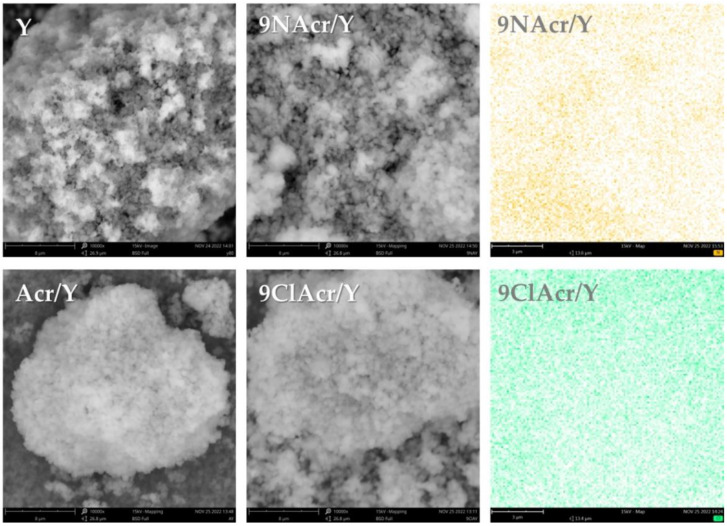
SEM micrographs (at 10,000×) of supported acridines and pristine Y zeolite with an EDX elemental map of nitrogen for 9NAcr/Y and chlorine for 9ClAcr/Y sample (at 20,000×).

Agglomerated clusters were observed in micrographs of starting Y zeolite with no clear morphological differentiation. However, a portion of cubic structures were present (Figure 1). The structure of the Y zeolite remained unaltered with the supporting of acridine (Acr) and its derivatives (9NAcr and 9ClAcr) homogeneously on the surface, as witnessed by EDS mapping of nitrogen and chlorine at drug-loaded zeolites, 9NAcr/Y and 9ClAcr/Y, respectively.

The Acr precursor and derived drug, 9NAcr and 9ClAcr (Figure S1) loadings were determined to be (17.9 ± 0.4) mg for Acr and moderately higher for (18.7 ± 0.5) mg for 9NAcr and (21.4 ± 0.5) mg for 9ClAcr samples per g Y zeolite.

### 3.2. Spectral Analysis

**Figure 2:**
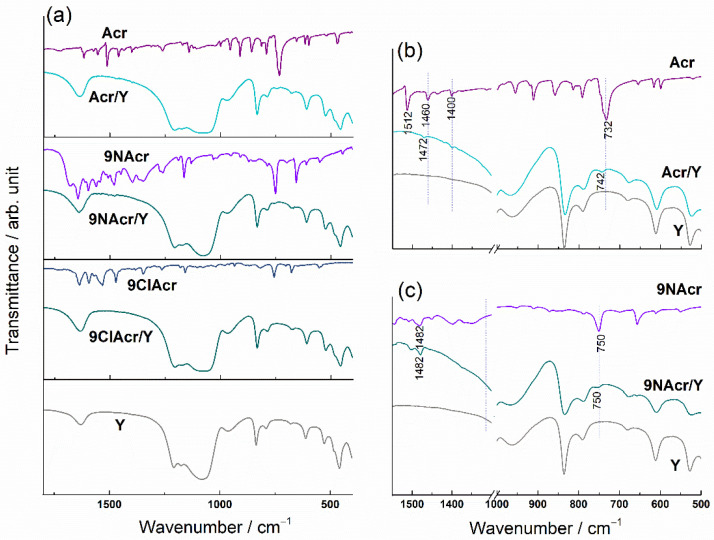
(**a**) FTIR spectra of pristine zeolite Y, acridines and supported samples, Spectral change induced by (**b**) Acr and (**c**) 9NAcr retention on the Y zeolite in the 1500–500 cm^−1^ range.

**Figure 3:**
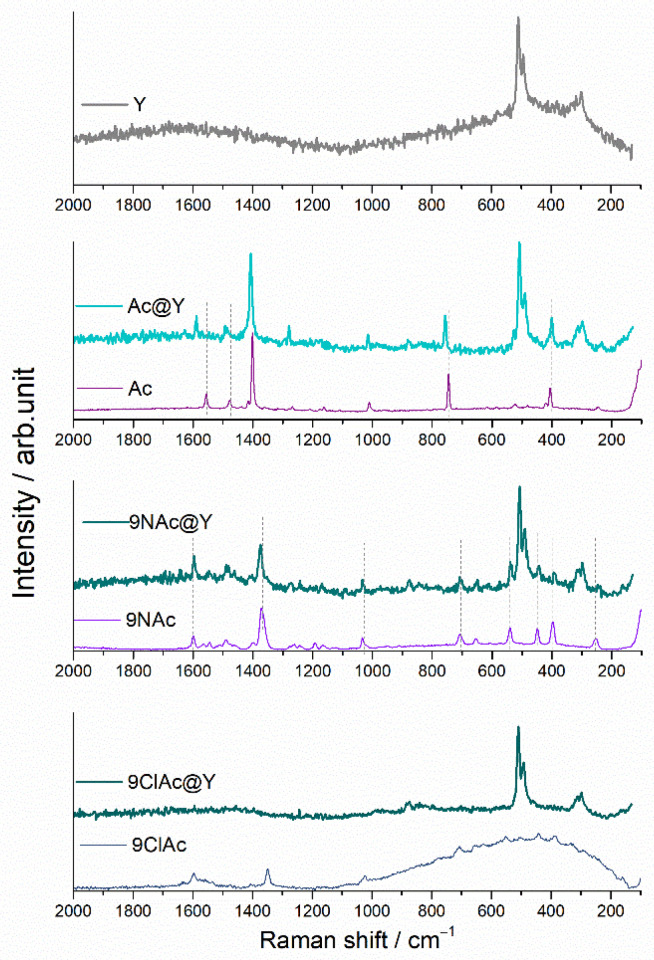
Raman spectra of pristine zeolite Y, acridines and supported samples.

To investigate the extent of the Acr and its derivatives interaction with Y zeolite, FTIR and Raman spectral analysis of post-loading (impregnated) and pristine zeolite samples were performed. For comparison, in Figure 2 and Figure 3, the spectra of Acr, 9ClAcr and 9NAcr of evaporated solutions used for impregnation are provided, too.

Flanigen described the classification of the infrared active zeolite vibrations as internal/external tetrahedral modes [32]. All vibrations of the Y zeolite are seen (without the change in bands positions and intensity ratio) in the Acr/Y, 9NAcr/Y and 9ClAcr/Y spectra, which confirms that drug impregnation leaves no change in the Y zeolite structure (Figure 2). Due to the low loadings of acridine and its derivatives in the samples, their bands are hardly distinguished in the FTIR spectra of the impregnated samples. This can be overcome, to some extent, by recording overdosed KBr discs (with a higher percentage of the sample mixed with KBr), Figure 2b.

A skeletal C-C stretching vibrations of benzene and pyridine units in Acr could be seen in the 1625-1475 region, while the remaining bands are ascribed to C-H vibrations [33]. The most intense acridine band, not overlapped with the zeolite bands, at about 732 cm^−1^, corresponds to C-H out-of-plane bending vibration, and it is visible in the spectrum of the impregnated zeolite sample Acr/Y, but it is shifted by 10 cm^−1^ towards higher wavenumbers. On the other hand, the bands of 9NAcr remain unaffected after impregnation on Y zeolite. 9ClAcr bands are not visible in the spectrum of 9ClAcr/Y, although EDS analysis confirmed the presence of homogeneously dispersed chlorine in this sample (Figure 1). The underlying cause is the overlapping of the most intense 9ClAcr vibrations by zeolite bands.

Stronger interaction of adsorbate molecules with zeolite carrier, evident for the sample Acr/Y, may induce alteration in charge distribution of molecule targeted for release from drug carrier. Due to the absence of strong interaction, retention of 9NAcr in its pristine form may allow its release as an unaltered structure.

Owing to the fact that Y zeolite has a moderate number of bands in the Raman spectrum, this method allows inspection of a number of acridine bands, which are free of overlapping with the zeolite bands (Figure 3). The spectrum of zeolite Y distinguishes vibrations at about 500 cm^−1^ and 300 cm^−1^, which are, respectively, attributed to the four- and six-membered rings (T-O-T, T = Al or Si) [34]. In all impregnated samples, these bands remain unchanged.

The Raman bands of acridine observed at 1556, 1478 and 1401 cm^−1^ are assigned to the ring stretching/v(C-C) modes, while in-plane C-H bending vibrations can be seen around 1010 cm^−1^. Skeletal vibrations/C-C bending is positioned below 800 cm^−1^ [35].

Raman spectra of Acr supported on zeolite exhibit band shifts towards higher wavenumbers in comparison to Acr band positions. The shortening of the bond indicates a tighter binding of Acr itself on Y zeolite relative to acridine derivatives. On the other hand, 9NAcr vibrations attain their positions upon loading onto zeolite.

From the aspect of prolonged drug release, it is beneficial to deliver unaltered drug molecules. Due to the intense 9ClAcr fluorescence, the Raman signal after software fluorescence correction is weak, and its bands are not visible in the spectrum of the 9ClAcr/Y.

### 3.3. Release Study

**Figure 4:**
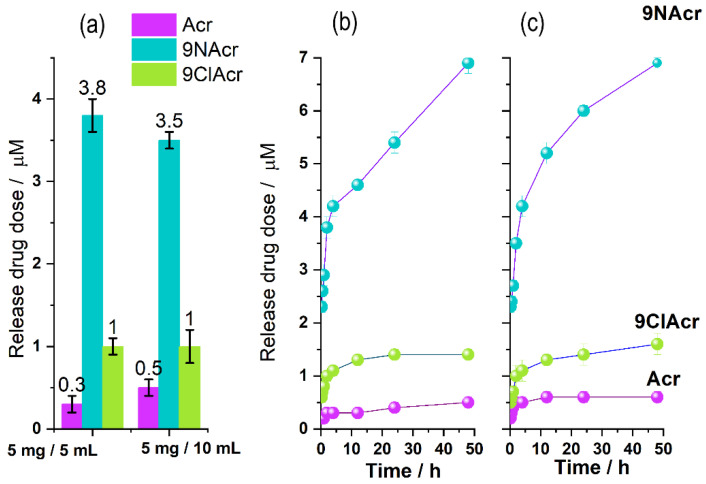
(**a**) A drug release quantification with respect to suspension solid/liquid ratios within a 2 h period, (**b**) the 48 h kinetics of the tested drugs release process within 1 mg/mL, and (**c**) 0.5 mg/mL loadings.

The drug release was performed in phosphate (pH 7.2) buffer solution with different loadings to test delivery performance within a 2 h period (Figure 4a). Delivered quantities rise in the Acr/Y–9ClAcr/Y–9NAcr/Y line. The notable result suggests that the concentration of the drug released is mainly independent of whether the experiment is performed in a 1 mg/mL or 0.5 mg/mL solid/liquid ratio, which indicates a steady release of the drug into available volume. This finding is associated with an even distribution of supported acridines, thus released quantity increases accordingly, providing the same released concentration. Sustained release is in line with the acridines’ limited water solubility and a higher extent of interaction with solvent molecules in a larger volume.

A three-dimensional tetrahedral zeolite framework allows each oxygen atom to be shared with two tetrahedra; thus, -Si-O-Si- network would be neutral. Each Si replacement with Al in the zeolite induces partial negative charges in the framework resulting in protonated -Si-OH-Al- groups that act as H-donor in hydrogen bonding. The distinct behaviour of drug-loaded zeolites in release tests can be explained by structural differences of tested acridines and respective pKa values. Acr acts as a weak base with a conjugate acid pKa value of 6.15. At pH, over its pKa (experiment pH = 7.2) the loss of hydrogen in the majority of the pyridine rings results in Acr partial release, while a substantial quantity remains attached to zeolite by hydrogen bonding with bridging hydroxyls in the zeolite. The slightly larger released amount of 9ClAcr, compared to Acr, stems from Cl-induced charge redistribution that disturbs the deprotonated pyridine nitrogen–zeolite interaction witnessed for pristine Acr precursor. Both Acr and 9ClAcr have one hydrogen bonding site and a topological polar surface area of 12.9 Å^2^ which account for their similar behaviour [36].

On the other hand, the highest 9NAcr release from loaded zeolite can point to the deciding effect of the amino groups, present along with pyridine nitrogens, resulting in one donor and two hydrogen bonding acceptor sites. The calculated pK value for 9NAcr is 9.3 for the pyridine nitrogen, while amino nitrogen is deprotonated over the entire pH range, retaining NH2 structure [29]. In addition, a topological polar surface area of 38.9 Å^2^ [36] enhances solvation and 9NAcr release, reaching up to 7 µM within 48 h (Figure 4a).

**Figure 5:**
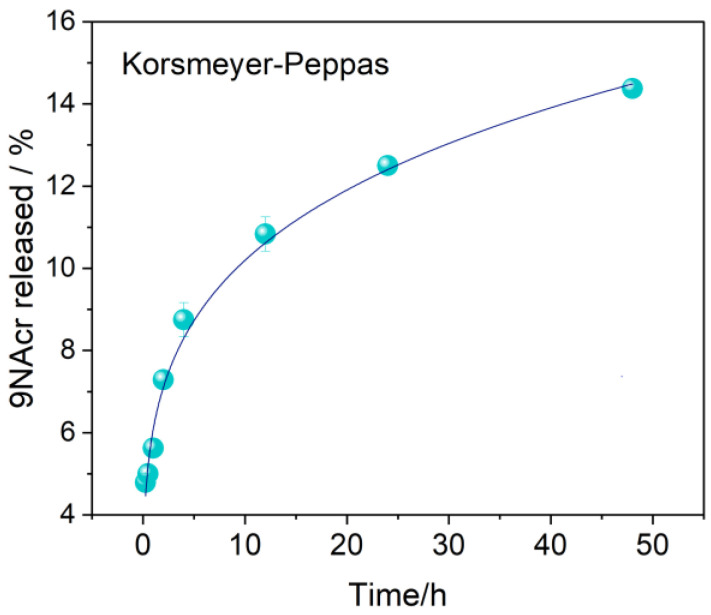
The drug release from the 9NAcr/Y sample fitting was performed using a Korsmeyer–Peppas model.

To acquire a definite release profile for investigated drugs, prolonged kinetics was monitored, confirming steady release (Figure 4b,c). The drug release profile from the 9NAcr/Y sample may be successfully fitted using a Korsmeyer–Peppas (KP) model with a correlation factor above 0.99 (Figure 5). Fitted parameters of release are exponent *n* = 0.224 ± 0.008, pointing to drug diffusion as the primary release mechanism, expected for non-swellable matrices, and often seen for zeolite carriers [3]. The rate constant is *k* = 0.061 ± 0.002 h − 0.024.

### 3.4. Optimization of Acridines and Zeolite Interactions

**Figure 6:**
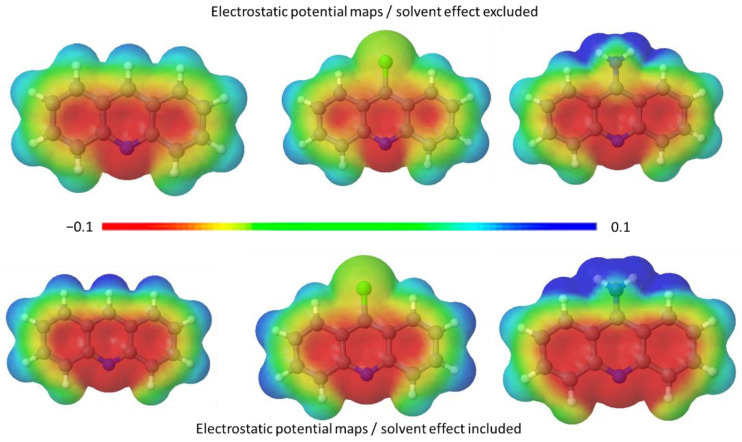
Acridine electrostatic potential maps with and without solvent effect.

**Figure 7:**
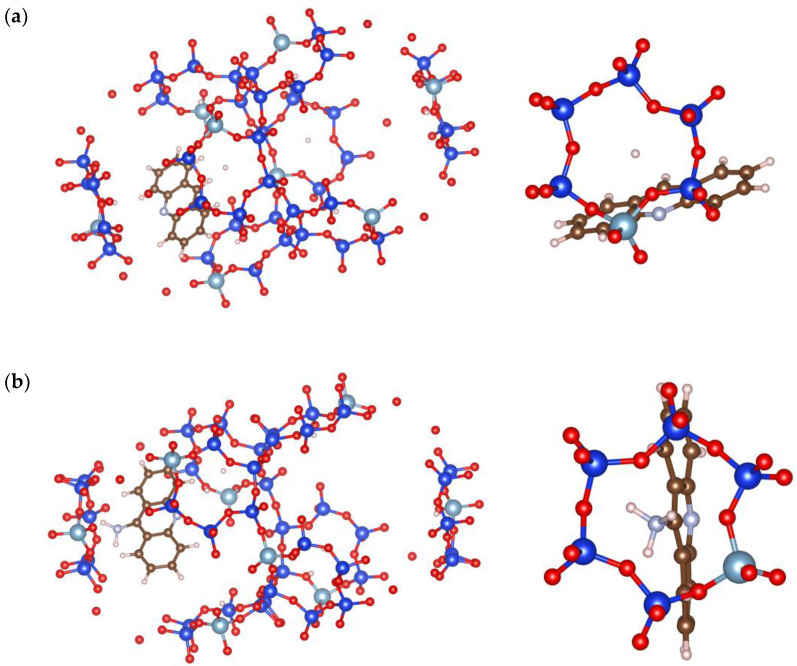
Optimized (**a**) Acr and (**b**) 9NAcr orientation within zeolite pores (**left**) and magnified view on the ring opening (**right**). Zeolite: O—red; Si—blue; Al—light blue (large spheres), 9NAcr/Acr: C—brown; N—light blue (small spheres); H—white.

Theoretical analysis and molecular optimisation with and without solvent effects are performed to estimate acridine charge distribution in detail (Figure 6) and orientation within zeolite pores (Figure 7).

There is a significant influence of aqueous surroundings on the electron distribution map of acridines. This is especially pronounced for 9NAcr, where the rise in the positive outer surface charge is concentrated on the amino group (Figure 6). The presence of solvent lowers the heat of formation for all molecules. The estimated value of solvation energy is −15.8 kcal/mol, −8.7 kcal/mol, and −6.3 kcal/mol for 9ClAcr, Acr, and 9NAcr, respectively (Table S1). System stabilization energy due to the presence of solvent is calculated to be approximately −0.4 eV for Acr and 9ClAcr and rises to around −1 eV for 9NAcr (Table S1), indicating its high hydrating affinity.

Calculations show that the charge distribution governs drug positioning within zeolite openings. Acr orientation (Figure 7a) is less centered and is positioned more toward the zeolite scaffold, with the pyridine core as the predominant center of interaction with the zeolite framework. In contrast, 9NAcr is positioned in the center of the zeolite pore, as the amino group of 9NAcr (Figure 7b) favours hydrogen bonding interaction with zeolite sites. This may support drug diffusion (Figure 5) as a release mechanism due to favourable orientation inside zeolite pores. The computed binding energy of acridines with Y zeolite is −55 kcal/mol for the Acr/Y system and −58 kcal/mol for 9NAcr/Y. Differences in calculated adsorption energies are negligible, in line with the comparable adsorption capacities for all tested molecules (18–21 mg/g). As 9NAcr is more energetically stabilized within the hydration sphere, its dissolution from the zeolite carrier (where it is partially desolvated and centered inside the pore opening) is more pronounced than for Acr and 9ClAcr molecules, as witnessed in the release experiment (Figure 4a).

### 3.5. Cytotoxicity of Supported Acridines

**Figure 8:**
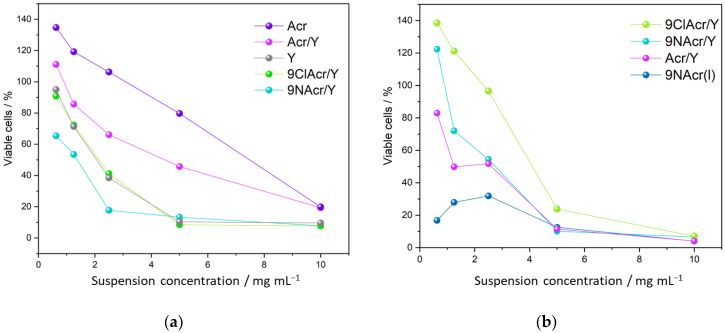
Cytotoxicity testing of selected supported acridines, Y zeolite and Acr on (**a**) HCT-116 cell line and (**b**) MRC-5 fibroblasts.

Finally, the cytotoxic effects of drugs released from the zeolites on the HCT-116 cancer cell line and MRC-5 fibroblasts were examined (Figure 8).

Prepared drug-loaded zeolite suspensions were tested in terms of their toxicity toward cancer cells (Figure 8a). A continuously decreasing dependence of cell viability on suspension concentration for all samples is recorded. Within the 48 h incubation period, we have witnessed Y zeolite boosting the cytotoxicity of Acr, as a drug itself exhibits relatively low toxicity toward cancer cells. However, supported Acr/Y remains less active than pristine Y zeolite over the tested concentration range. The zeolite-supported amino and chloro-derivatives impose higher toxicity than the Acr/Y sample. This may be anticipated considering the low release of Acr from zeolite at pH 7.2.

The highest cytotoxic impact, up to 5 mg/mL, is witnessed for 9NAcr/Y, delivering up to 7 µM concentration in 0.5–1 mg/mL suspensions (Figure 8a). This concentration fits well with previous findings documenting the anticancer activity of 9-acridinyl amino acid derivatives in the targeted µM range—IC50 was 6 μM for the lung carcinoma cell line [15]. Guo et al. investigated the HCT-116 cell line and p110g protein suppression by 9NAcr treatment. Different 9Acr doses and treatment periods led to downregulation of protein expression even at 4–8 h post-treatment time with a threshold at 5 µM 9Acr [37], which corresponds to our cytotoxicity findings. Similarly, acriflavine may also be incorporated along with 5-Fluorouracil chemotherapeutics for colorectal carcinoma treatment to boost efficiency [38]; either alone or combined, these compounds require 7.4–11.6 µM concentration to reach IC50 value.

The anticancer activity of acridines has recently been summarized in [39]. The action of acridine derivatives may interrupt DNA synthesis by intercalating in DNA thus inhibiting topoisomerase II/I [40]. Wang et al. reported that acridine derivatives stabilize p53 and induce p53-related cell apoptosis [16]. Moreover, amino acridines exert cytotoxic and antiangiogenic actions through the modulation of radical oxygen species and cytokine levels [17].

The aluminium in the zeolite framework enables its ion-exchange properties, which, in turn, enable interfering with intracellular pathways causing cytotoxicity/necrosis [41]. Moreover, the formation of phagosome witnessed for FAU type zeolite used for the treatment of cancer cells is especially pronounced for this zeotype in comparison to other frameworks. Since phagosome formation is regarded as the initiation of cell death [42], this may be a reasonable explanation of zeolite cytotoxic action.

The cytotoxic effect of supported acridines on HCT-116 cells reveals that the zeolite carrier improves toxicity towards cancer cells. The synergetic effect of both potent components, drug and selected zeolite, support add to the performance witnessed for each component.

To test whether supported acridines exert a favourable action on fibroblasts, a comparison of selected results is given in Figure 8b. Interestingly, findings suggest that Acr derivatives on Y zeolite allow higher fibroblasts viability in contrast to Acr/Y at low drug loadings, opposite to relations seen for cancer cells. Contrary to supported drugs tested in cell medium, the 9NAcr(l) supernatant solution stands out as the most cytotoxic formulation among tested compounds. This is the reason why its impregnation on the solid carrier may be beneficial to healthy tissue while adequately affecting cancer cells viability.

## 4. Conclusions

A systematic investigation comprising spectral, microscopic, theoretical and cytotoxic evaluation of the zeolite Y utilisation in supporting and release of acridines (Acr, 9-ClAcr and 9-NAcr) is performed.

The highest Acr loading reached 17.9 mg/g, while for 9-ClAcr and 9-NAcr, the retained quantity was 21.4 mg/g and 18.7 mg/g, respectively. Supporting acridines on zeolite did not induce morphological changes in zeolite, while EDS elemental maps confirm their even distribution. After the drug loading onto zeolite, their steady release was monitored in the 48 h timeframe. Regardless of the selected suspension loadings, 9-NAcr showed the best release efficiency, with a maximum concentration of 6.9 µM. In the case of Acr and 9-ClAcr, slightly lower delivered quantities were detected due to the lower extent of hydrogen bonding. Insight into interactions in the delivery system was finally resolved by molecular optimization in an aqueous environment. The presence of solvent lowers the heat of formation for all acridines estimating the value of solvation energy to −15.8 kcal/mol, −8.7 kcal/mol, and −6.3 kcal/mol for 9ClAcr, Acr, and 9NAcr, respectively. The proposed mechanism revolves around the amino group hydrogen bonding potential of 9-NAcr, not seen with Acr and 9-ClAcr, accounting for its highest stability and release quantity. Spectroscopic analysis documented band shifting towards higher wavenumbers for Acr relative to its amino derivatives. This beneficial finding supports unaltered acridine derivatives release from the zeolite carrier.

The novel drug delivery system released necessary cytotoxic drug concentrations in the µM range. Cytotoxicity investigation was in line with the confirmed steady release of the supported drugs in the 48 h period. The cytotoxic effect of supported acridines on HCT-116 cells reveals that the zeolite carrier improves toxicity towards cancer cells. In the range of concentrations up to 5 mg/mL, the highest efficiency was shown by zeolite-impregnated 9-NAcr. On the contrary, acridine derivatives on Y zeolite induce higher fibroblast viability than Acr. The deciding result is reflected in supported vs. liquid drug toxicity. While testing in the fibroblast cells medium, the 9-NAcr solution stands out as the most cytotoxic formulation among tested drugs. Consequently, 9-NAcr delivery via zeolite carrier favours healthy tissue preservation while accompanying increased toxicity toward cancer cells.

The obtained results resolve the benefits of a zeolite-supporting procedure for acridine and its derivatives in cancer treatment, with particular attention drawn to 9-NAcr performance and potential for further testing.

## Data Availability

Data are contained within the article or Supplementary Materials.

## Supplementary Materials

The following supporting information can be downloaded at: https://www.mdpi.com/article/10.3390/jfb14030173/s1, Figure S1: MolView presentation and assessment of acridines dimensions; Table S1: Solvation enthalpy for tested acridines calculated from heat of formation with and without solvation sphere.
