# Supplementary material for: Can Zeolite-Supporting Acridines Boost Their Anticancer Performance?
Source: J Funct Biomater. 2023 Mar 22;14(3):173. doi: 10.3390/jfb14030173 (PMC10056282; doi:10.3390/jfb14030173)
Supplement: Supplementary file 1 [file jfb-14-00173-s001.zip › jfb-2276252-supplementary.pdf]

Supplementary Materials

Can zeolite-supporting acridines boost their anticancer performance?

Maja Ranković[a], Anka Jevremović[a], Aleksandra Janošević Ležaić[b], Aleksandar Arsenijević[c], Jelena Rupar[b], Vladimir Dobričić[b], Bojana Nedić Vasiljević[a], Nemanja Gavrilov[a], Danica Bajuk-Bogdanović[a], Maja Milojević-Rakić[a]

[a]University of Belgrade–Faculty of Physical Chemistry, 11000 Belgrade, Serbia.

[b]University of Belgrade–Faculty of Pharmacy, Department of Physical Chemistry and Instrumental Methods, 11221 Belgrade, Serbia

[c]University of Kragujevac–Faculty of Medical Sciences, Department of Pharmacy and Center for molecular medicine and stem cells research, 34000 Kragujevac, Serbia

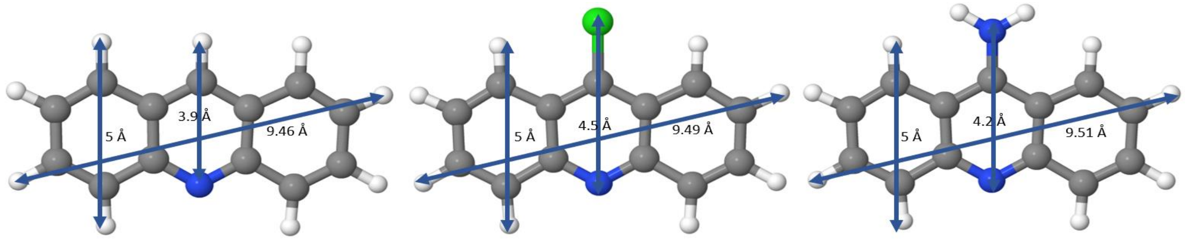

Figure S1. MolView presentation (H – white, C – grey, Cl – green, N – blue) and assessment of acridines dimensions.

Table S1. Solvation enthalpy for tested acridines calculated from heat of formation with and without solvation sphere.

	**Heat of formation/kcal per mol**		
	Acr	9ClAcr	9NAcr
**No solvation**	71.7	62.5	69.2
**Solvation**	63.0	56.2	53.4
**Dielectric part / eV**	-0.44	-0.34	-1.03
